# Informed consent and ethical re-use of African genomic data

**DOI:** 10.1186/s40246-014-0018-7

**Published:** 2014-10-22

**Authors:** Galen EB Wright, Adebowale A Adeyemo, Nicki Tiffin

**Affiliations:** 1 South African National Bioinformatics Institute/Medical Research Council of South Africa Bioinformatics Unit, University of the Western Cape, Private Bag X17, Bellville 7535, South Africa; 2 National Human Genome Research Institute, National Institutes of Health, Building 12A, Room 4047, 12 South Drive, MSC 5635, Bethesda 20892-5635, MD, USA

**Keywords:** Informed consent, Secondary use, Ethics, Africa

## Abstract

Rapid advances in human genomic research are increasing the availability of genomic data for secondary analysis. Particularly in the case of vulnerable African populations, ethics and informed consent processes need to be transparent¿both to ensure participant protection, as well as to share skills and to evolve best practice for informed consent from a shared knowledge base. An open dialogue between all stakeholders can facilitate this.

## Ethical re-use of African genomic data

The health genomics revolution is well under way, thanks to the increased accessibility of rapidly advancing sequencing technologies [1]. Africa is primed to enter this arena, especially given significant new research support from the Human Heredity and Health in Africa (H3Africa) Initiative [2] funded by the National Institutes of Health (NIH, USA) and the Wellcome Trust (UK) [3]. Within Africa, the support from this initiative is unprecedented in size and scope, currently including eight consortia/collaborative centers, eight research projects, four biorepository projects and a continent-wide bioinformatics network. Genomic data-sharing and secondary use is the key to accelerating discovery and many initiatives such as the Global Alliance for Genomics and Health [4], and the H3Africa Bioinformatics Network [5] are enabling data-sharing. This must, however, be realised within a framework of high ethical standards and truly informed participant consent [6]-[8].

Consent for secondary use of data and samples is sensitive within Africa, for various reasons: localised cultural sensitivities, and the vast cultural and ethnic diversity across African populations, are not always appreciated by external researchers [9]; many African populations are vulnerable to exploitation because of low education levels, and poor access to health care may create dependence on study inclusion for access to care [10],[11]; and African governments, institutes, and researchers are wary of repeating the historical outflow of samples and data from the continent through collaborations outside Africa [12],[13].

Institutional and governmental ethics review boards (IRBs) are currently the gate-keepers for re-use of African genomic data and for validating informed consent processes. There are many excellent IRBs and ethicists in Africa, but they are often under-resourced (for example, see [14] and [15]). They may lack capacity to train members in evaluating human genomics research, e.g. issues with genomic data de-identification,¿a conceptual paradigm shift away from existing clinical and genetic data that do not encapsulate the individual as a whole. There is, therefore, a strong need in Africa for ethics skills development [16], sharing of ethics and informed consent experiences, and building on current practice. Ensuring robust informed consent processes is crucial for protection of African research participants¿particularly because once data leaves Africa under secondary use consent, it moves beyond the jurisdiction of the country of origin and participant protection becomes reliant on the host country.

To date, we have found it surprisingly difficult to access informed consent templates, standard operating procedures and `information for participants¿ from published African genomic studies [13]. Reluctance to share ethics and informed consent template documentation may arise from concerns around privacy and legal implications for both IRBs and researchers. In the absence of such prior information, each new genomic study often has to develop anew the informed consent process, templates, and forms without being able to learn from previous studies. Through this continual reinvention of the informed consent process, we fail to grow our understanding of beneficence and risks for African research participants or to improve these processes incrementally with each new study.

Issues around data re-use and informed consent may raise concerns about secondary use of participant genomic data. Currently, a statement about ethics approval and informed consent is usually provided in publications and is often considered sufficient for secondary use access to study data obtained through correspondence with the authors. In some cases, data access committees may review proposals for secondary use of genomic data (for example, see [17]), but the original ethics documentation and informed consent templates are seldom available for scrutiny by downstream data users to ensure that participant consent was fairly and ethically obtained for their own secondary analysis. Similarly, except for some large-scale projects of human genetic variation [8], such resources are seldom available for reference by researchers developing their own best practice for informed consent.

These issues raise questions around transparency and skills-sharing to develop the best practice for ethics, informed consent and participant protection particularly in Africa¿although they are pertinent for all health genomics research.

Firstly, can journals that publish genomic research involving human subjects encourage more transparency through submission of ethics-related documentation and templates when accepting manuscripts for publication? This might take the form of providing the option to submit ethics documentation templates (such as informed consent templates) for some studies or encouragement to provide such documentation as supplementary data where authors are willing to do so. Secondly, can public databases for human genome data facilitate transparency around ethics and informed consent processes? Full ethics documentation could be supplied at the time of data submission and could be provided to researchers requesting access to the data through appropriate committees. Thirdly, should the human genomic research community explore ways (e.g. through centralized databases) to share templates for informed consent and patient information in the interests of ethics skills-sharing and developing best practices for participant protection? Such resources could assist researchers in developing appropriate ethics templates for their own studies (but should not be seen as developing a `one-size-fits-all¿ approach to informed consent protocols).

Whilst the answers remain unclear, there is a need for open dialogue between stakeholders about maintaining and supporting sustainable ethical compliance and integrity when dealing with human genomic data. As long as economic and participant vulnerability for African populations remains, these issues are particularly pertinent when dealing with data originating from Africa.

## Abbreviations

H3Africa: Human Heredity and Health in Africa

H3ABioNet: H3Africa bioinformatics network

IRB: institutional review board

NIH: National Institutes of Health

## Competing interests

The authors declare that they have no competing interests.

## Authors¿ contributions

The authors all planned, wrote and reviewed the manuscript. All authors read and approved the final manuscript.
